# What has changed in the outbreaking populations of the severe crop pest whitefly species in cassava in two decades?

**DOI:** 10.1038/s41598-019-50259-0

**Published:** 2019-10-15

**Authors:** Hadija M. Ally, Hajar El Hamss, Christophe Simiand, M. N. Maruthi, John Colvin, Christopher A. Omongo, Helene Delatte

**Affiliations:** 1 Université de La Réunion, 97715 15 Avenue René Cassin, Sainte-Clotilde, La Reunion France; 2 CIRAD, UMR PVBMT, 7 Chemin de l’Irat, Ligne Paradis, 97410 Saint Pierre, La Reunion France; 3 Tanzania Agricultural Research Institute-Ukiriguru, P.O. Box, 1433 Mwanza, Tanzania; 4 Natural Resources Institute (NRI), University of Greenwich, Central Avenue, Chatham Maritime, Kent, ME4 4TB UK; 5 Root Crops Programme, National Crops Resource Research Institute (RCP-NaCRRI), P.O. Box, 7084 Kampala, Uganda

**Keywords:** Ecology, Molecular biology

## Abstract

High populations of African cassava whitefly (*Bemisia tabaci*) have been associated with epidemics of two viral diseases in Eastern Africa. We investigated population dynamics and genetic patterns by comparing whiteflies collected on cassava in 1997, during the first whitefly upsurges in Uganda, with collections made in 2017 from the same locations. Nuclear markers and mtCOI barcoding sequences were used on 662 samples. The composition of the SSA1 population changed significantly over the 20-year period with the SSA1-SG2 percentage increasing from 0.9 to 48.6%. SSA1-SG1 and SSA1-SG2 clearly interbreed, confirming that they are a single biological species called SSA1. The whitefly species composition changed: in 1997, SSA1, SSA2 and *B. afer* were present; in 2017, no SSA2 was found. These data and those of other publications do not support the ‘invader’ hypothesis. Our evidence shows that no new species or new population were found in 20 years, instead, the distribution of already present genetic clusters composing SSA1 species have changed over time and that this may be in response to several factors including the introduction of new cassava varieties or climate changes. The practical implications are that cassava genotypes possessing both whitefly and disease resistances are needed urgently.

## Introduction

Crop protection involves practices to manage the plant diseases, weeds and pests that damage agricultural crops and forestry. It plays a key role in safeguarding global crop production against losses, thereby helping to meet the increasing demand for food caused by a growing human population^1^. Cassava (*Manihot esculenta* Crantz) is an important root crop, which is drought tolerant and able to grow under suboptimal conditions such as low soil fertility^2^. It provides food for about 800 million people worldwide^3^. Cassava has proven to be an invaluable food security crop, particularly to smallholder farmers in Sub-Saharan African countries^4^. Cassava production, however, has been decreasing, particularly in East Africa, despite the increasing area under cultivation^5^. The main cause of this trend is two major viral diseases, cassava mosaic disease (CMD) and cassava brown streak disease (CBSD). These are both transmitted by their whitefly vector, *Bemisia tabaci* (Gennadius) (Hemiptera: Aleyrodidae)^6^.

CMD in Africa is caused by at least seven species of single-stranded DNA cassava mosaic begomoviruses (CMBs) (family Geminiviridae: genus *Begomovirus*)^7,8^ and CBSD by two cassava brown streak ipomoviruses (CBSIs) (family Potyviridae: genus *Ipomovirus*)^9–12^. In addition to being transmitted by *B. tabaci*, both CMBs and CBSIs are spread by farmers, through the use of virus-infected stem cuttings. The two diseases can occur singly or in dual infections in cassava and the damage can be severe. Yield losses of about 47% from CMD infected fields were recorded in eastern and central African cassava-growing areas^13^, while in other fields losses of up to 70% were reported due to CBSD^14^.

*B. tabaci* has recently been considered as a complex of species, including at least 35 morphologically indistinguishable species^15^. Members of the different species are found on more than 500 plant-host species in 74 families, which includes crops that are crucial to food security, such as cassava^15–17^. These pests affect plants by direct phloem feeding by nymphs and adults on crop foliage or production of honeydew, which encourages the growth of sooty mould fungus on leaves^18,19^. However, by far the greatest impact is caused by the spread of >350 plant viruses including CMBs and CBSIs^20–22^. Epidemics of CMD and CBSD have been reported in several parts of Eastern and Southern Africa since the early 1990s and these were associated with unusually high numbers of whiteflies on cassava^21,23,24^. The presence of these ‘superabundant’ populations has been responsible for the rapid spread and development of two disease epidemics^25^, but the reason(s) for their upsurges remain uncertain^26^.

Five putative species of *B. tabaci* (described by their mtCO1 marker) have been found colonising cassava in sub-Saharan Africa (SSA) and these were named serially SSA1 to SSA5^16,25,27,28^, with several sub-groups reported for some species. The SSA1 species is widely distributed in Africa; SSA2 is mostly found in the eastern, southern and central areas of Africa as well as in the south of Spain; while SSA3 and SSA4 have been reported in Cameroon and the Central African Republic; SSA5 has only been described in the Ivory Coast and South Africa^27–29^. SSA2 was hypothesised to be an invasive species associated with the CMD epidemic in Uganda in the 1990s, but has subsequently been rarely found^30,31^. In addition, phylogenetically distinct populations have been described within the SSA1 species, known as SSA1 sub-groups 1 and 2 (SSA1-SG1 and SSA1-SG2, respectively), which were also associated with the CMD and CBSD epidemics^25,26,32^.

Analysis of genotypes and genetic diversity of *B. tabaci* species is of crucial importance as it can facilitate selection of appropriate management control measures^33^. Analysing the nuclear genetic diversity of whitefly populations had been performed in the past using several types of markers, among which neutral-codominant markers such as microsatellites gave reliable results. Those markers allowed to distinguish *B tabaci* species and populations within those species, including Med Q1 and ASl^34^, Med and MEAM1^35–37^, Med Q1/Q2^38^, or between a wide range of species worldwide^39^. Nevertheless, those markers had not been commonly used to untangle population structure among SSA species in Sub-Sahara Africa.

The objectives of the current study, therefore, were to understand: (i) whitefly species’ distributions in cassava fields in Uganda in 1997 during the initial stages of the CMD epidemic and compare these with the high whitefly populations still present in 2017; (ii) the genetic pattern (diversity and genetic structuring) of population dynamics over two decades in rapidly evolving *B. tabaci* species. To meet these objectives, we estimated the genetic diversity and population structuring of the whitefly species by sequencing the partial mitochondrial cytochrome oxidase I (mtCOI) barcoding region and 13 nuclear markers from specimens collected in 1997 and 2017 from the same geographical location.

## Results

### Whitefly abundance and CMD and CBSD symptoms

Whitefly abundance varied between fields from <10 to over 500 adults per plant. Fields with over 100 adult whiteflies per plant were considered superabundant populations. Based on this criterion, eight (61.5%) of the 13 fields visited in 2017 had superabundant whitefly populations, among which two fields did not show CMD and CBSD symptoms (Table 1). The distribution of CMD within fields was higher (69.2%) than that of CBSD (46.2%) with a maximum leaf severity score of 4 detected in two fields. Moreover, 38.5% of the fields were dually infected with both viruses. Furthermore, during the 1997 survey, three fields (42.9%) had superabundant whitefly populations (Table 1) and one of these had plants exhibiting severe CMD symptoms. Three fields had plants with CMD symptoms, despite a low whitefly number (<10 per plant). No CBSD symptoms in any plants were recorded during 1997.

**Table 1:** Location and information of adult whiteflies collected in Uganda.

FN	Village name	DN	SY	CA	CV	WC	CMS	CBS	X	Y
F1	Mityana I	Mityana	1997	—		—	2	—	*	*
F1	Kireku	Mityana	2017	7	Gomboka	100	3	1	N00.43564	E032.04041
F2	Masaka 25	Mpigi	1997	—		100	3	—	*	*
F2	Kalagala	Mpigi	2017	6	Akena	100	1	3	N00.00979	E032.00677
F3	NaCCRI	Wakiso	2017	2.5	NASE 3	1	1	1	N00.51831	E032.63553
F5	Kampala-Masaka 55 km	Kalungu	1997	—		100	1	—	*	*
F5	Kyanagolo	Kalungu	2017	4	TME 14	1	4	3	S00.16989	E031.83412
F6	After Nkosi 15 km	Masaka	1997	—		—	1	—	*	*
F6	Masaka	Masaka	2017	6	Unknown	10	3	3	S00.33294	E031.70984
F7	Kalisizo	Rakai	2017	6	Unknown	100	2	2	S00.52627	E031.64813
F8	Masaka 50 km	Rakai	1997	—		—	1	—	*	*
F8	Kiwesi	Rakai	2017	3	TME 204	10	2	2	S00.66515	E031.53927
F9	Rutula	Rakai	2017	4	TME 14	10	1	1	S00.69034	E031.43948
F10	Nabigasa	Rakai	2017	7	Kalandila	100	3	1	S00.89538	E031.44637
F11	Agasamvu	Rakai	2017	6	TME 14	500	4	2	S00.98063	E031.41873
F12	After Nkosi	Kalungu	1997	—		100	1	—	*	*
F12	Ntale	Kalungu	2017	3	TME 14	500	1	1	S00.12179	E031.75773
F13	Mityana II	Gomba	1997	—		—	2	—	*	*
F13	Wasinda	Gomba	2017	5	NASE 3	100	1	1	N00.17379	E031.92822
F14	NaCRRI Valley	Wakiso	2017	8	NAROCAS 2	100	3	1	N00.52556	E032.62680

Field number (FN), village name, district where sample population was collected (DN), year of sampling (SY), samples were made in February 1997 and February 2017), cassava age (CA), cassava variety (CV), whitefly count (CV), CMD and CBSD severity symptoms scores (CMS and CBS) and GPS coordinates.

^*^Exact GPS coordinates are not available for the 1997 survey; sites were referenced as distances from Kampala on different roads.

### Phylogenetic analysis

The partial mtCOI gene of 665 whiteflies was sequenced, of which 219 were from the 1997 collection (110 eggs, 79 nymphs, 28 pupae and 2 adults) and the remaining 446 were all adults from the 2017 collection. After manual checking and editing for errors, the mtCOI sequences were trimmed to different lengths: 700 bp (*n* = 251), 657 bp (*n* = 219), 500 bp (*n* = 112) and 300 bp (*n* = 80) depending on the sequence quality obtained. Despite the shorter sizes of some sequences (300 bp), it was possible to differentiate between putative species and SSA1 sub-groups, as well as to use the shorter sequences as species tags in further analysis. To increase robustness of the phylogenetic tree, a total of 470 sequences that were at least 651 bp long, together with an additional 12 sequences from the GenBank, were used. Bayesian phylogenetic analysis used to generate the tree divided our sequences into four main clusters (SSA1(-SG1 -SG2 and -SG3), SSA2, Mediterranean (Med) and *Bemisia afer*) supported by high posterior probability values (>0.9) (data not shown). The specific group of haplotypes within SSA1 named SSA1- SG1 were found to be dominant with 183 individuals (84%), followed by SSA2 (*n* = 22, 10%), whereas others belonged to the other haplotype groups named SSA1-SG2 (*n* = 2, 0.9%) and SSA1-SG3 (*n = *1, 0.5%), and to *B. afer* individuals (*n* = 10, 4.6%) from the 1997 collection (Supplementary Fig. S1). The 2017 samples revealed both SSA1-SG1 (*n* = 126, 50.2%) and SSA1-SG2 (*n* = 122, 48.6%) as the dominant groups over all observed genetic clusters. The other species characterised were Med and *B. afer*, which together represented 1.2% of the total (Supplementary Fig. S2).

**Figure 1:**
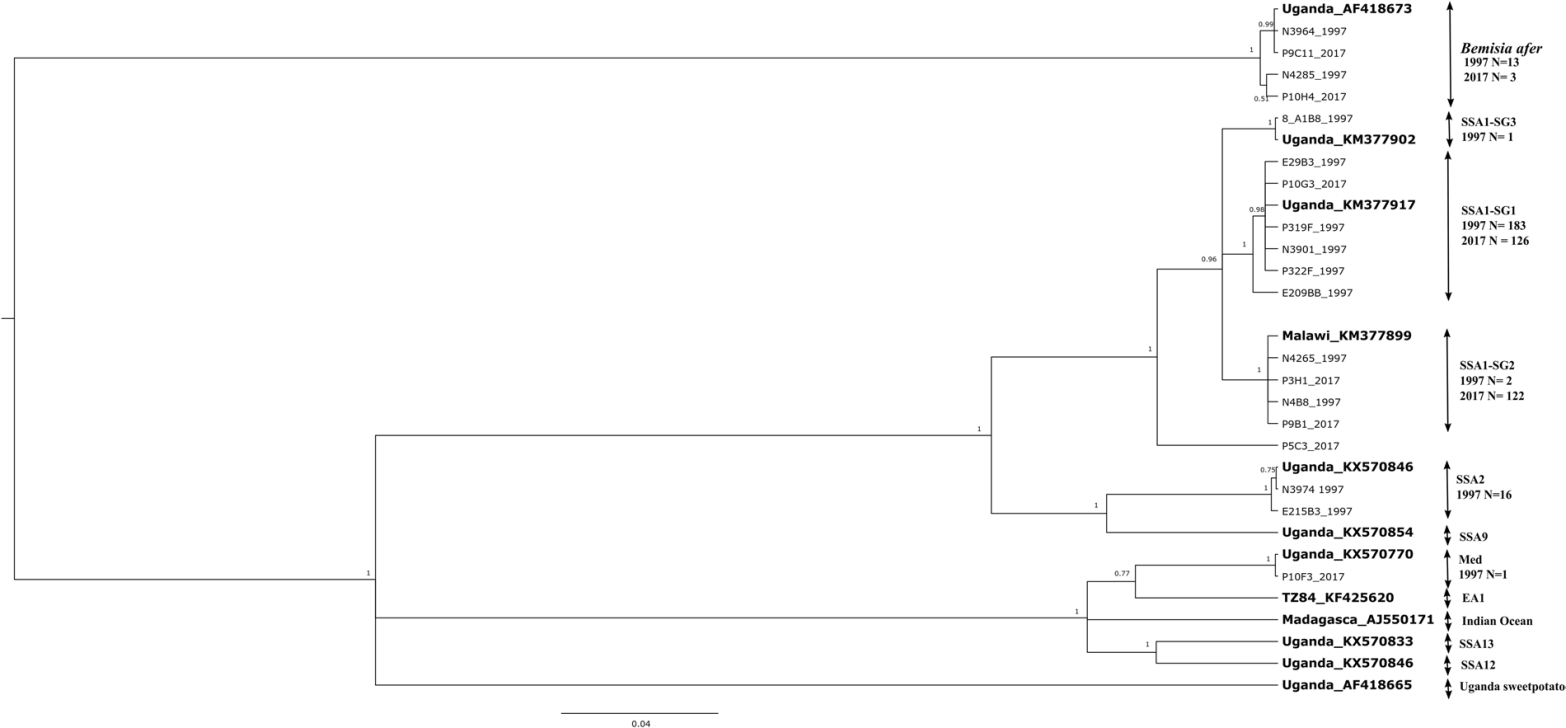
Rooted posterior probability phylogenetic tree generated by MrBayes using the Markov chain Monte Carlo method for all the different mtDNA COI haplotype sequences (651 bp) of 1997 and 2017 samples together (*n* = 14) with reference sequences (*n* = 12, in bold) obtained from GenBank for comparison. Numbers associated with nodes indicate the posterior probability for those nodes. Horizontal bars represent genetic distances as indicated by the scale bar, vertical distances are arbitrary.

Haplotype diversity results revealed 12 haplotypes from *B. tabaci* species within the combined dataset of longest sequences (651 bp), comprising 470 (Table 2) individuals (219 and 251 from 1997 and 2017 respectively); however, only two of these were observed in both 1997 and 2017 (Fig. 1). Ten haplotypes were observed in 1997, among which five were observed for SSA1-SG1, with the largest group containing 176 individuals (84.2%) represented as P319F in the phylogenetic tree (Fig. 1). This haplotype shared 100% identity with the previously identified sequence of KM377899^40^ and KX570785^41^, both from Uganda. The remaining four SSA1-SG1 haplotypes contained nine individuals (4.3%). Two haplotypes were observed for SSA1-SG2. Apart from SSA1, two haplotypes were found for SSA2 (*n* = 22, 10.5%) from 1997 collected samples. In 2017, five haplotypes were found from *B. tabaci* species, including one haplotype for SSA1-SG1 (*n* = 126, 50.6%) represented as P10G3 (Fig. 1). These individuals shared 100% identity with the majority of SSA1-SG1 found in 1997. In addition, three SSA1-SG2 haplotypes were observed, among which 117 individuals (47%) shared 100% identity with the KM377899^40^ and KX570790^41^ reference sequences recognised in Malawi and Uganda obtained from GenBank, and the other two SSA1-SG2 haplotypes contained five individuals (2%). One haplotype (*n* = 1, 0.4%) of the Med species was found. Apart from 12 haplotypes of *B. tabaci* species, two other *B. afer* haplotypes were also observed. All the new haplotypes were submitted to GenBank and were assigned accession numbers from MK360160 to MK360177 (Table 2).

**Table 2:** Haplotype distribution within fields F1–F14 (Table 1) and two different years (1997 or 2017).

Field no.	Year	Species status	Ni (mtCOI)	SP code (mtCOI)	SR	Accession
F1	1997	Nymph	2	SSA2	**N3974**	**MK360171**
	1997	Nymph (20), pupa (12)	32	SSA1-SG1	N4155	Same haplotype as MK360162
	1997	Nymph	1	SSA1-SG1	**N3901**	**MK360172**
	1997	Nymph (7), Egg (1)	6	*B. afer*	**N3964**	**MK360160**
	1997	Nymph	1	SSA1-SG2	**N4265**	**MK360170**
	1997	Nymph	2	*B. afer*	**N4285**	**MK360169**
	2017	Adult	19	SSA1-SG1	P9G3	Same haplotype as MK360164
	2017	Adult	13	SSA1-SG2	**P3H1**	**MK360168**
	2017	Adult	2	SSA1-SG2	**P9B1**	**MK360167**
	1997	Egg	18	SSA2	E213B3	Same haplotype as MK360171
	1997	Egg	2	SSA2	**E215B3**	**MK360174**
	1997	Egg	1	SSA1-SG1	**E29B3**	**MK360176**
F2	1997	Egg	1	*B. afer*	E14B3	Same haplotype as MK360160
	2017	Adult	15	SSA1-SG1	P9D4	Same haplotype as MK360164
	2017	Adult	17	SSA1-SG2	P3H9	Same haplotype as MK360168
	2017	Adult	2	SSA1-SG2	P9B3	Same haplotype as MK360167
	2017	Adult	11	SSA1-SG1	P9D4	Same haplotype as MK360164
F3	2017	Adult	3	SSA1-SG2	P9G5	Same haplotype as MK360168
	1997	Egg	17	SSA1-SG1	E208BB	Same haplotype as MK360162
F5	1997	Egg	1	SSA1-SG1	E233BC	Same haplotype as MK360176
	2017	Adult	5	SSA1-SG1	P9E7	Same haplotype as MK360164
	2017	Adult	9	SSA1-SG2	P9C8	Same haplotype as MK360168
	1997	Egg	22	SSA1-SG1	E31B6	Same haplotype as MK360164
F6	1997	Egg	1	SSA1-SG1	E27B6	Same haplotype as MK360172
	2017	Adult	5	SSA1-SG1	P9B9	Same haplotype as MK360164
	2017	Adult	5	SSA1-SG2	P9G8	Same haplotype as MK360168
	2017	Adult	3	SSA1-SG1	P9B10	Same haplotype as MK360164
F7	2017	Adult	11	SSA1-SG2	P9G10	Same haplotype as MK360168
	2017	Adult	1	*B. afer*	**P9C11**	**MK360166**
	1997	Egg	28	SSA1-SG1	E96B8	Same haplotype as MK360162
	1997	Egg	2	SSA1-SG1	E111B8	Same haplotype as MK360176
F8	1997	Nymph	1	SSA1-SG2	**N4B8**	**MK360173**
	1997	Adult	1	SSA1-SG3	**A1B8**	**MK360177**
	2017	Adult	7	SSA1-SG1	P10D1	Same haplotype as MK360164
	2017	Adult	4	SSA1-SG2	P10F2	Same haplotype as MK360168
	2017	Adult	5	SSA1-SG1	P10A3	Same haplotype as MK360164
F9	2017	Adult	10	SSA1-SG2	P10B3	Same haplotype as MK360168
	2017	Adult	4	SSA1-SG1	**P10G3**	**MK360164**
	2017	Adult	8	SSA1-SG2	P10E3	Same haplotype as MK360168
F10	2017	Adult	1	*B. afer*	**P10H4**	**MK360163**
	2017	Adult	1	Med	**P10F3**	**MK360165**
	2017	Adult	5	SSA1-SG1	P10B6	Same haplotype as MK360164
F11	2017	Adult	9	SSA1-SG2	P10F5	Same haplotype as MK360168
	1997	Egg (17), adult (1)	18	SSA1-SG1	E205BB	Same haplotype as MK360162
F12	1997	Egg	1	SSA1-SG1	**E209BB**	**MK360175**
	2017	Adult	12	SSA1-SG1	P10C8	Same haplotype as MK360164
	2017	Adult	10	SSA1-SG2	P10C7	Same haplotype as MK360168
	1997	Nymph (43), pupa (15)	59	SSA1-SG1	**P319F**	**MK360162**
	1997	Pupa	1	SSA1-SG1	**P322F**	**MK360161**
F13	1997	Nymph	1	*B. afer*	N4326	Same haplotype as MK360160
	2017	Adult	13	SSA1-SG1	P10G9	Same haplotype as MK360164
	2017	Adult	15	SSA1-SG2	P10C10	Same haplotype as MK360168
F14	2017	Adult	22	SSA1-SG1	P8C10	Same haplotype as MK360164
	2017	Adult	4	SSA1-SG2	P10A11	Same haplotype as MK360168
Total			470			

**Figure 2:**
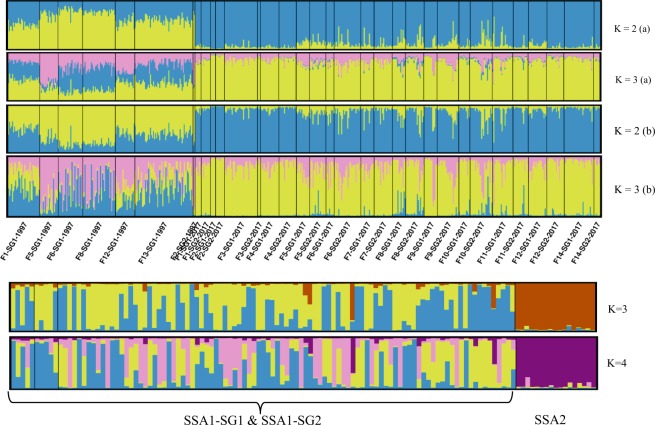
STRUCTURE bar plots for SSA1 and SSA2 populations collected from Uganda (**a**) for 33 populations of SSA1 arranged by subgroup, site and year at *K* = 2 and 3, e.g., K2(a) and K3(a) with recessive allele option turned on, and K2(b) and K3(b) without the option turned on. (**b**) For 102 randomly selected SSA1-SG1 and SSA1-SG2 together with 17 individuals of SSA2 at *K* = 3 and 4. The black line within SSA1 separates individuals of SSA1-SG1 and SSA1-SG2 for 2017 and SSA1-SG1 for 1997.

Species status, numbers in parenthesis represent number of individuals at each stage; total number of individuals amplified for mtCOI (Ni); species code according to mtCOI barcoding (SP code); Individual code for selected representative among similar mtCOI haplotype sequences (SR), where bold individuals were used in the construction of the phylogenetic tree (Fig. 2b) and sequences were submitted to GenBank; and Accession refers to the accession number given by GenBank.

### Nuclear genetic analysis

A total of 594 out of 662 individuals (407 and 203 from the 2017 and 1997 samples, respectively) were successfully genotyped. All loci were checked with Microchecker^42^ and no PCR artefacts linked to large allele drop-out or stuttering were detected. All individuals and loci with missing data greater than 20% and/or 25% of null alleles were discarded from the dataset, meaning that 68 individuals and one loci (CIRSSA41) were removed. The number of alleles per locus from the 13 microsatellite markers over the whole dataset ranged from 5 to 52. The highest polymorphism observed was for the P5 locus and the lowest was for the CIRSSA2 locus. The mean null allele frequency for all loci and populations was 0.128 but ranged from 0.01 to 0.56 (from CIRSSA6 to CIRSSA41, respectively) (Table 3).

**Table 3:** Characteristics of loci used for nuclear analysis.

LN	Reference	Sequence name	Motif	NuA	FL	%MS	NA	Range (bp)
MS145	Dalmon *et al*.^36^	F: CCTACCCATGAGAGCGGTAA R: TCAACAAACGCGTTCTTCAC	(AC)9	0.24	PET	11	29	124–278
P59	Delatte *et al*.^53^	R: TTTGCCAACTGAAGCACATCAATCA	(T)44(G)18	0.17	6-FAM	0.8	16	152–216
P7	Delatte *et al*.^53^	F: AGGGTGTCAGGTCAGGTAGC R: TTTGCGTAATAGAAAA	8(GT)	0.16	VIC	6.1	40	105–261
WF2H06	Hadjistylli *et al*., 2014	F: TATTCGCCAATCGATTCCTT R: CGGCGGAAATTTCGATAAA	(TTTG)11	0.12	NED	6.8	24	102–208
P62	Delatte *et al*.^53^	F: CTTCCTTAGCACGGCAGAAT R: TTTGGCGCAATTTTTAGCGTCTGT	(GT)8	0.1	6-FAM	5.4	49	112–296
WF1G03	Hadjistylli *et al*., 2014	F: CTCCAAAATGGGACTTGAAC R: GTAGAAGCCACACATACTAGCAC	(GTTT)8	0.07	PET	4.5	29	100–292
WF1D04	Hadjistylli *et al*., 2014	F: GTTGTTAGGTTACAGGGTTTGTC R: GTCTTTACTTCTTTTCCTCCG	(CAAA)16	0.06	VIC	1.2	19	100–182
P5	Delatte *et al*.^53^	F: ATTAGCCTTGCTTGGGTCCT R: TTTGCAAAAACAAAAGCATGTGTCAAA	(GT)8	0.16	NED	15.5	52	100–282
CIRSSA2	This study	F: ACAATGCATGTTGATTGTGAA R: TGAAAATGTCTACGGCCAGA	(AG)6	0.01	VIC	0.3	5	100–108
CIRSSA6	This study	F: CATATCGGTCATTATCCGCA R: CATCAGGCTGGAAAGACGAG	(TC)6	0.11	VIC	0.2	8	125–173
CIRSSA7	This study	F: TGGCGATCCTCTTCTTGTTT R: AAGAAGCAGCAGTTCATCCG	(TC)5	0.13	PET	0.6	8	134–152
CIRSSA13	This study	F: AGTGCTGAAGGTCCACCGTA R: GGGATTTCCAGGGGTTAAGA	(CT)6	0.03	NED	1.4	7	203–225
CIRSSA41	This study	F: TGGGTGCATGGTTCTTACAG R: TATCCGGTCGACAAACACAA	(CT)6	0.56	6-FAM	57	15	210–267

Locus name (LN), source reference, sequence name, microsatellite repeat motif, null allele frequency in the whole dataset (NuA), fluorochromes used for PCR product detection (FL), percentage of missing data in the whole dataset (%MS), number of alleles counted per locus in the whole dataset (NA), allele size range (Range, bp). Loci CIRSSA2, CIRSSA6, CIRSSA7, CIRSSA13 and CIRSSA41 are described here for the first time. The MS, NA, range and null allele columns were obtained on the SSA1 and SSA2 populations sampled.

The population genetic diversity indices were calculated in SSA1 and SSA2 species separately. The results from SSA1 species showed the mean alleles richness over all loci, per field, ranged from 4.22 (*n* = 14; 2017) to 6.66 (*n* = 57; 1997) with the highest mean values observed in the 1997 collection (Supplementary Table S1). None of our collected samples, analysed per population, showed deviation from Hardy–Weinberg equilibrium, using the exact test of Markov Chain of Monte Carlo (MCMC) (Supplementary Table S1). Three out of 66 pairs of loci across all populations showed deviation from genotypic disequilibrium after Bonferroni correction.

### Genetic structuring and population differentiation

Bayesian clustering analysis revealed three major genetic clusters from the SSA1 population with an optimal number of clusters of *K* = 3 (estimated by means of ∆*K*, as described by Evanno *et al*.^43^). The first two genetic clusters at K3, in Fig. 2b, dominated the 1997 *B. tabaci* samples. Individuals of the two mtCO1 sub-groups SSA1-SG1 and SSA1-SG2 were not differentiated and belonged to the same genetic clusters (Fig. 2b). The third genetic cluster, denoted by yellow colour, dominated the 2017 collections except for some individuals of the two other genetic clusters found within the 1997 samples at *K* = 3. Conversely, a few individuals assigned to the pink genetic clusters were also found in 2017 collections of both yellow and blue genetic clusters. We can also observe a proportion of individuals with less than 50% of posterior probability assigned to one genetic cluster, which were perceived to be part of several genetic clusters. These individuals could be assigned as individuals with gene flow between genetic clusters.

Although there were few samples of the SSA2 population (17 individuals), another Bayesian analysis was run together with SSA1 individuals to understand the genetic pattern between the two-putative species. To decrease the effect of unbalanced samples, 102 samples of SSA1 together with 17 samples of SSA2 were randomly chosen, and similar results were obtained showing some level of shared genetic background, as expected for closely related species (Fig. 2b).

**Figure 3:**
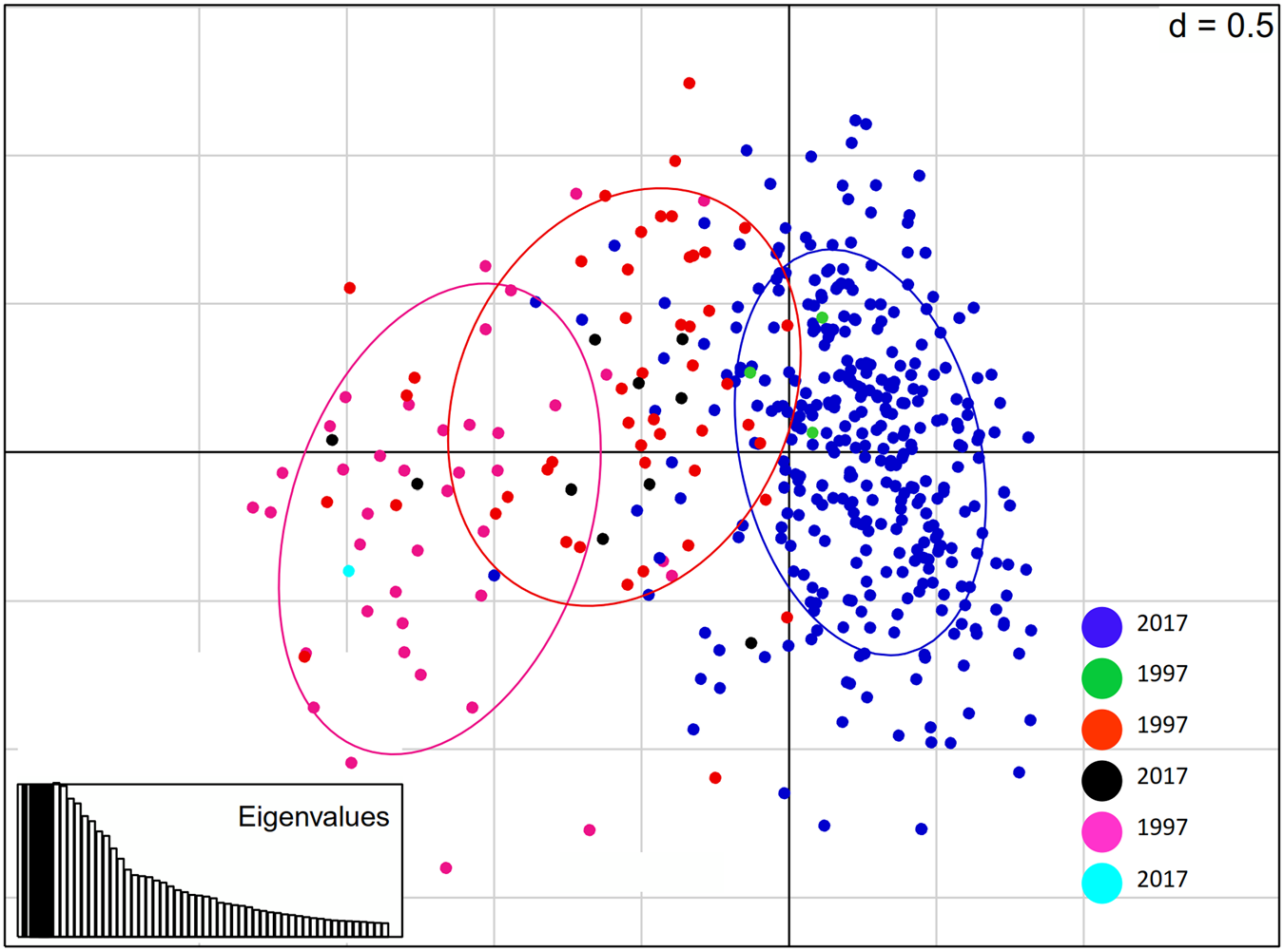
Principal component analysis of *B. tabaci* (SSA1-SG1 and SSA1-SG2) populations from Uganda. Colours show the genetic clusters found with the Bayesian analysis of structure at *K* = 3. Each dot represents one individual. The pink cluster is dominated by the 1997 population, whereas the blue and orange clusters are dominated by the 2017 population. In each cluster there are few individuals of different years represented by green, brown and black dots.

Individuals with 70% posterior probability from the Bayesian analysis dataset were selected and used to perform a principal component analysis (PCA), subsequently the analysis split the dataset into three clusters/ellipses similar to the previously identified genetic clusters of Bayesian analysis (Fig. 3). The 2017 samples were aggregated in one ellipse. In contrast, the majority of 1997 individuals belonged to the other two ellipses. However, some individuals from the two collections were mixed within clusters, for instance, with individuals in green, cyan and black. In parallel, a discriminant analysis of principal components (DAPC) was performed and the best BIC value was found at *K* = 3, as the best *K* number of assumed populations. Accordingly, the DAPC spread the dataset into three clusters; two were dominated by 1997 collections, whereas the third cluster was represented by the 2017 samples (Data not shown).

The AMOVA carried out on our SSA1 samples to test for population differentiation between years, using only sites that were sampled in 1997 and 2017 (ie. 6 sites were considered for each year) showed a very low, but significant variation (Table 4).

**Table 4:** Analysis of molecular variance (AMOVA average over loci) from Ugandan populations of *B. tabaci*, comparing SSA1 populations between sampling year, *indicates significant variation among populations within species and within individuals.

Source of variation	Sum of square	Sum of component variation	Percentage of variation	Fixation indices
Among years	76.16	0.19310	4.99843	FCT: 0,05*
Among population within years	88.96	0.09658	2.50012	FSC: 0,03*
Within individuals	2374.86	3.57350	92.50145	FST: 0,07*
Total	2539.98	3.86318		

Further analysis of differentiation between groups was performed through analysis of the population pairwise matrix of genetic distances Fst between sites, subgroups and years. Results revealed a few significant differences between SSA1-SG1 and SSA1-SG2 of the 2017 samples for only 33 comparisons. Most of the populations of 1997 were significantly different from the ones of 2017, except for field 5 (Supplementary Table 2).

The bottelneck analyses performed on the 2017 dataset showed that all populations had undergone a significant bottleneck (One-tailed Wilcoxon sign-rank tests, P < 0.05) in the recent past with the SMM model (Supplementary Table 3).

## Discussion

We used two different molecular markers to identify population genetic variations within the African *B. tabaci* colonizing cassava in Uganda, to compare 2017 populations with those of the 1997 outbreak. Our results reveal that SSA1 was the dominant species in Uganda both in 1997 and 2017 and that its subgroups SG1 and SG2 can interbreed. Populations within SSA1 were found to be structured into three genetic clusters, irrespective of subgroups, which varied in abundance between 1997 and 2017. The SSA2 individuals were clustered separately. The main results obtained here are showing that the genetic composition of SSA1 whitefly species has changed rapidly over the 20 years period, which is contrasting with the previous invader hypothesis.

Out of the 13 cassava fields visited in 2017, we observed 8 fields with >100 mean adult whiteflies per plant, which were defined as having superabundant populations. Within those eight fields, 63% (*n* = 5) showed CMD symptoms and 50% showed symptoms of CBSD. The association between the two diseases and *B. tabaci* population on cassava has been reported by Colvin *et al*. and Legg *et al*.^21,44^. In the remaining five fields with <50 mean adult whiteflies per plant, up to 60% CMD and CBSD symptoms were observed. The improved cassava varieties TME14 and TME 204 were grown in these fields. Despite the relatively low number of whiteflies observed in the field located at Kynagolo (1–9 mean adult whiteflies per plant) planted with TME 14 and the field at Masaka (10–49 mean adult whiteflies per plant) planted with an unknown variety, both had average CMD and CBSD symptom severity (Table 1).

Regardless of the superabundant whitefly population in two fields (Ntale and Wasinda), no CMD and CBSD symptoms were observed, which may be related to the type of variety grown (NASE3, an improved cassava variety, which might be tolerant to CMD and CBSD).

The average age of cassava field with high whitefly abundance (100 and above) was 6 months, which contrasts with the age recently shown by Kalyebi^45^ of 2–3 months. These observed discrepancies might be linked to the surveyed cassava varieties, which might be more whitefly susceptible in our case, or the very small amount of field samples in our study (*n* = 8). Furthermore, our study revealed whitefly abundance increased toward the southern part of Uganda, with a maximum population of >500 whiteflies per plant in 2017. This result corresponds to the previous studies conducted in Uganda during the 1990s^46,47^.

Despite all efforts made to combat CMD and CBSD diseases since the first outbreaks of whitefly populations reported in the 1990s^23,46,48^ in Uganda, most of the cassava varieties grown in the 2017 surveyed fields (TME 14, TME 204 and NAROCAS II) were infected with both diseases. Development of whiteflies on cassava is the result of synergistic interaction of several factors including viruses, bacterial symbionts and cassava genotypes^49^, all of which require more research attention. In order to combat these problems effectively, research and development efforts need to be focussed on creating cassava varieties that combine both virus- and whitefly-resistance traits.

SSA1-SG1 was present at all sites in 1997 and 2017 (Table 2). The presence of SSA1-SG1 in different regions of central and eastern Africa, including Uganda, has been reported in several studies. Interestingly, a balanced distribution of SSA1-SG1 and SSA1-SG2 was found in 2017 compared with the very low proportions of SSA1-SG2 in 1997 (0.9% in 1997 vs 48.6% in 2017). Recent studies describing the SSA1 sub-groups found similar results in Uganda^25,30,50^.

The proportion of SSA2 was low (10%) in 1997 and we did not detect it at all in 2017. Legg *et al*.^25^ also reported a drastic decline in SSA2 from 63.9% for 1997–1999 sampling to 1.4% by 2009–2010. The reduction of SSA2 in favour of SSA1-SG1 and SSA1-SG2 in Uganda has also been reported in two recent studies^30,51^. The reason for the decrease of SSA2 in eastern Africa is unknown but could be associated with less suitable environmental conditions, such as the use of improved cassava varieties following the CMD epidemic, which might have impacted the abundance of SSA1. In addition, it might be related to biological consequences of mating interruption between the two species, where copulation events occur between individuals of the two species but without viable progeny. Consequences of this behaviour is observed with a decreasing success of mating of the species in lower abundance^15,52^.

Analysis of mitochondrial DNA placed SSA1-SG1 and SSA1-SG2 in different haplotype groups within SSA1; however, nuclear analyses based on several methods revealed substantive gene flow between these two haplotypes. The ability of these two groups to interbreed and exchange genetic material resulted in there being no significant genetic differentiation between the individuals of both groups. The homogenisation of this group from potentially two different maternal lineages probably resulted in the maintenance and increase of SSA1-SG2 over the years. Similar results were observed from a genomic approach, showing no differences between SSA1-SG2 and SSA1-SG1 from Burundi, Tanzania, Rwanda or the Democratic Republic of Congo^31^. These results collectively indicate that SSA1-SG2 and SSA1-SG1 should not be considered as different entities, but only as different mitochondrial haplotypes within the SSA1 species.

Nuclear analyses from 1997 sampling also revealed that SSA2 individuals analysed were considered as a separate group to SSA1; however, with a signal of low shared background between both groups. This weak signal could be explain by the fact that both species are closely related ones. Similar results were seen in the recent genomic analysis by Wosula *et al*.^31^ and had been observed in La Réunion between the invasive Middle East Asia minor 1 (MEAM1) and indigenous Indian Ocean (IO) species^53^. High abundant SSA1 populations and low abundant SSA2 populations in sympatry might have created conditions favouring mating between those groups, which could have resulted in few cases of mating success between both cryptic species.

Our study also revealed no new populations between 1997 and 2017, but a significant genetic difference between the two collection periods. This is clearly shown in the Bayesian analysis structure (Fig. 2a) with the dominance of one cluster at K3 for 2017 SSA1 populations, whereas the two other genetic clusters characterised the 1997 SSA1 populations. Despite the variations in distribution of genetic clusters, we also observed mixed genetic patterns within populations between the years.

The Bayesian analyses, DAPC (BIC criteria) and PCA all showed structuring of SSA1 putative species into three genetic clusters that can interbreed. However, despite the gene flow patterns between several individuals, this structuring into three genetic clusters was stable between 1997 and 2017 and the individuals did not completely homogenise into a single population, with significant differentiation observed between populations of both years. Although the reasons for this are not entirely clear, mating preferences or other specific loose barriers to hybridisation may act to support this pattern. Presence of different symbiont communities could also be a factor. Indeed, some symbionts are known to play such a role in other insects^54,55^ as well as whiteflies, where a specific bacterial community (*Arsenophonus* and/or *Cardinium*) had been partly implicated in manipulating reproduction of MEAM I and IO species^56^. SSA1 supports a high bacterial diversity^51,57^; however, no link has yet been established between the complex bacterial community and hybridisation barriers. Further studies should be conducted to better understand the roles of endosymbionts in different *B. tabaci* species.

Despite the moderate null allele frequencies detected in our dataset, our results remained consistent through the four different analyses. All analyses produced similar patterns, which indicates the robustness of our results. All results obtained here categorically reject the hypothesis that new outbreaks of whiteflies in Uganda in the 1990s were due to the arrival of a new population or species of whiteflies^25^. Nevertheless, frequency of specific genetic clusters significantly changed over the studied 20-year period within the SSA1 species, with 2017 populations having a strong signature of a recent bottleneck event. It is possible that the most abundant genetic cluster comprising SSA1-SG1 and SSA1-SG2 might have overcome or displaced the previous most abundant genetic cluster. Correspondingly, three new hypotheses might be raised to explain the observed results: (i) the previous “old” dominant genetic cluster might be less fit for new cassava cultivars released in Uganda, (ii) environmental change occurred within the studied 20-year period and the SSA1-SG1 and SSA1-SG2 clusters were preferably adapted to it and (iii) the re-emergence of CBSD in Uganda in the early 2000s^58^. Confirming these hypotheses will require further experiments.

The strategy of using disease-resistant cultivars has not proved effective in combating CMD and CBSD. Some ‘improved’ varieties such as TME 14 and TME 204 became susceptible in Uganda^3^ and many virus-resistant cassava varieties are highly susceptible to whiteflies^59^. The intensification of cassava production to meet the high demand for food under increasing human population in the era of climate change might be impossible without the concomitant control of *B. tabaci* populations and development of virus-resistant crop varieties. This can only be achieved by a better understanding of the main viral vectors, which will facilitate design and selection of appropriate disease management and control measures.

## Methods

### Whitefly collection

**Figure 4:**
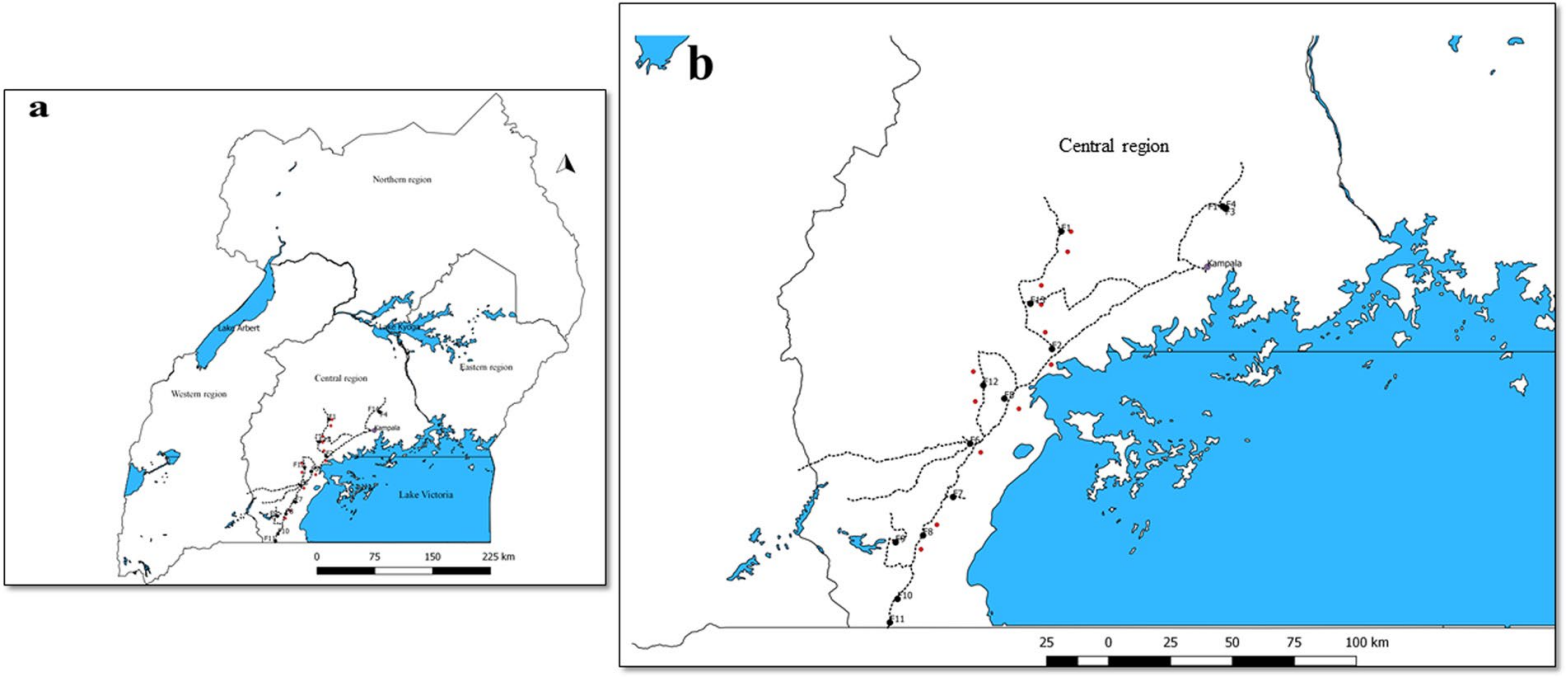
Geographical locations of sampling surveys conducted in (**a**) Uganda as a whole and (**b**) part of the central region in which sampling was conducted. Red and black circles are sample sites for whitefly collections made in February 1997 and February 2017.

Live adult whiteflies were collected from cassava plants during a survey of 13 fields from seven districts (Mityana, Mpigi, Wakisa, Kalungu, Masaka, Rakai and Gomba) in Uganda in February 2017 (Table 1, Fig. 4). The fields were separated by about 20 km (except for three that were <10 km apart) and their GPS coordinates were recorded. QGIS v.2.18.17 online software (https://qgis.org) was used to map the site locations (Fig. 4). Whiteflies were collected with a mouth aspirator and then preserved in Eppendorf tubes containing absolute ethanol. In the same geographical location adult whiteflies and cassava leaves with eggs, nymphs and pupa were collected (Table 1, Fig. 4, within 2–3 km radius) in February 1997 and stored at −80 °C. No GPS coordinates were recorded in 1997 but the names of sites/villages and recorded distances were used to approximately match the old and new sites.

### Determination of whitefly population

The number of adult whiteflies on the top five leaves of five plants selected randomly in each cassava field was recorded as described by Sseruwagi *et al*.^60^ in the 2017 sampling. The number of whiteflies per plant was estimated according to the following system: “1” = 1–9 adults per plant, “10” = 10–49, “50” = 50–99, “100” = 100–499 and “500” = >500.

### Assessment of CMD and CBSD symptom severity

The symptom severity for CMD and CBSD were recorded for each sampled field. The severity was assessed by using a disease scale 1–5 according to Sseruwagi *et al*.^60^, where 1 = no disease symptoms and 5 = the most severe symptoms. Five plants were randomly assessed in each field in 2017.

### DNA extraction of *B. tabaci*

Leica MZ8 stereomicroscope 100X (Leica Microsystems, Nanterre, France) was used for selection, at most 35 adult female whiteflies were selected from each field. A total of 662 samples were successfully extracted for DNA in this study (108 eggs, 78 nymphs, 28 pupae and 2 adult whiteflies from the 1997 collection, and 446 adults from the 2017 collection). Sex differentiation for eggs, nymphs and pupae was obtained indirectly, with the use of the microsatellite markers, whenever all loci were homozygotes for a given individual it was considered as a male. Two methods of DNA extraction were utilised. The non-destructive method was used for 2017 samples at 3P, CIRAD UMR PVBMT in Reunion Island, as described in Delatte *et al*.^61^ and the destructive method of Ghosh *et al*.^51^ for 1997 collections at Natural Resource Institute (NRI), University of Greenwich, England.

### Mitochondrial DNA amplification and sequencing

A total of 662 individuals were successfully PCR-amplified and sequenced for mtCOI by using a primer pair designed by Mugerwa *et al*.^41^ (2195Bt 5′-TGRTTTTTTGGTCATCCRGAAGT-3′ and C012/Bt-sh2 5′-TTTACTGCACTTTCTGCC-3′). The PCR reaction mixture was prepared with a final volume of 15 µl, containing 7.5 µl of Type-it (2x) PCR master mix (Qiagen, France), 4.1 µl of pure HPLC water CHROMASOLV (Sigma-Aldrich, France), 0.8 µl of each primer and 1 µl of DNA template. Initial denaturation of DNA template occurred at 95 °C for 15 min followed by 40 cycles of denaturation at 95 °C for 30 s, primer annealing at 52 °C for 30 s, extension at 72 °C for 1 min and final extension at 72 °C for 10 min. Plates were sent to the Macrogen Europe laboratory for sequencing.

### Sequence analysis

Sequences were manually edited and aligned using the Geneious R10 software^62^. The number and distribution of haplotypes within our sequences were achieved through DnaSP v.6 software^63^. The selected sequences together with reference sequences from the literature were aligned using ClustalW^64^ before being subjected to Jmodeltest 2.1.10^65^. The phylogenetic tree was computed using MrBayes^66^ at GTR + G (the closest to the selected model under MrBayes). Four Markov chains were conducted simultaneously for 1 100 000 generations starting from random initial trees, and sampled every 200 generations. Variation in the ML scores was examined graphically and 10% of the trees generated prior to stabilization of ML scores were discarded.

### Microsatellite PCR amplification and genotyping–Microsatellite design

Two pools of extracted DNA of 25 individuals (each tube) of *B. tabaci* from laboratory colonies of SSA2, SSA1-SG1, SSA1-SG2, SSA1-SG3 and SSA3 were made and sent to GenoScreen_VR_ (Genoscreen, Lille, France). Each pool contained 10.2 ng of DNA. The company developed a microsatellite-enriched library using a 454GS-FLX Titanium pyrosequencing^67^ tool. The enriched library was then constructed as described by Atiama *et al*.^68^. Total DNA was enriched by probes with the following motifs: TG, TC, AAC, AGG, ACAT, ACG, AAG and ACTC. About 534,451 reads were obtained with average fragment length of 247 bp. A first filter of quality was applied to discard short fragments (<40 bp) and low-intensity fragments, which removed 38% of the sequences.

The software QDD^69^ was run on the remaining sequences to identify microsatellite motifs in 73,060 raw sequences, among which 160 primers were designed. The objective was to obtain primers that would cross-amplify between all the pooled species and other whiteflies from the same complex of species with different sizes. From these, 41 primers were selected that could amplify various fragment lengths (100–260 bp) and had different repeat motifs (from di nucleotides to tetra nucleotide motifs). Those primers were tested by PCR individually, on four female specimens of the following species or whitefly genotype groups (-SG): SSA1-SG1, SSA1-SG2, SSA1-SG3, SSA2, SSA3, MEAM1, Med and IO. The amplified DNA was loaded on agarose gels and sorted. Among all those tested primers we kept nine primers that were (i) amplifying for all species or genotype groups with good signal intensity, and (ii) giving polymorphisms between individuals within species and between species/genotypes. These nine primers were fluorescently labelled (forward primer; Applied Biosystems, Waltham, MA, USA) and tested in simplex and multiplex PCR mixes on several field samples of the different whitefly species named above. Only five of them were retained in the present study, the other four were discarded due to the high number of null alleles observed in different populations and species within the species complex tested.

### Amplification and genotyping of old and recent field populations from Uganda

PCR for genotyping was conducted using 13 microsatellite loci, which were combined in three multiplex primer reactions. Five of the markers were newly developed for this study (Table 2). All markers were selected based on their ability to amplify different species within *B. tabaci* complex.

A PCR mix of 15 µl was prepared with 7.5 µl of 2x multiplex PCR master mix (Type-it, QIAGEN), 4.5 µl of HPLC water and 0.1 µl of each primer followed by addition of 2 µl of template DNA. The volume was slightly changed in Mix 2, in which 0.2 µl of WF1GO3 and P5 primers were used. All PCR programs were as follows: initial denaturation 95 °C (15 min) followed by 40 cycles, 95 °C (30 s), 55 °C (180 s), 72 °C (1 min) and at 60 °C (15 min) for denaturation, primer annealing, extension and final extension, respectively, except for Mix 3 for which the annealing temperature was increased from 55 °C to 56 °C. Prior to genotyping, the amplified PCR products were diluted in different ratios according to the band intensity obtained for each mix. The final mix consisted of 10.8 µl of formamide, 0.2 µl of Applied Biosystems LIZ size marker and 1 µl of diluted amplified DNA. The mix was run in an Applied Biosystems 3130XL DNA sequencer machine. Genotypic data were retrieved visualised and scored manually using Gene mapper v.4.0 software.

### Population structure analysis

The Bayesian cluster approach with Structure v.2.3.4^70^ was used to assess genetic population structure between individuals. The method assigns individuals to different clusters (a series of K to be set). Each K is the number of estimated population clusters characterised by posterior probabilities. Structure 2.3.4 was set at 100,000 burn in length with run length of 1,000,000 MCMC, this step was repeated three times and K was set to range from 1 to 20. The dataset was arranged according to mtCOI results and field numbers. The best number of clusters (K) was estimated by means of ∆K as described by Evanno *et al*.^43^ using the online program Structure Harvester^71^. An online program CLUMPAK (Clustering Markov Packager Across)^72^ was used to summarise the best K posterior probabilities and to reconstruct the bar plots using Clumpp^73^ and Distruct^74^ software.

As null alleles were still recorded in our datasets, we then ran two Bayesian analyses using 12 microsatellite loci with and without the recessive alleles option, as explained by Falush *et al*.^75^. Both datasets were executed using burn-in length of 100,000 and MCMC run length of 1,000,000, repeated three times, and an assumed number of population (*K*) values between 1 and 20. Similar results were obtained from both analyses (Fig. 2a,b), showing robust analyses regardless of null alleles.

### Population genetic analyses

The basic population parameters were analysed by using a set of programs within Genetix v.4.05.2, such as the number of alleles per population, expected heterozygosity and observed heterozygosity (according to the method of Nei^76^) and correlation within individuals following the method of Weir and Cockerham^77^. Deviation from Hardy-Weinberg equilibrium was tested using MCMC (run length of 1,000,000) implemented in Arlequin v.3.5.2.2^78^ following the method utilized by Guo and Thomson^79^. GENEPOP v.4.2^80^ was used to test genotypic disequilibrium by Fisher’s method^81^. The effect of null alleles on inferring population structure was studied, as described by Falush *et al*.^75^. Allelic richness using rarefaction was estimated by FSTAT v.2.9.3.2^82^. Genetic differentiation among year, between populations within year was inferred by AMOVA by Arlequin. PCA and DAPC were also used to determine the genetic clusters among individuals using R software v. 3.4.2^83^ with the Adegenet package^84^.

Recent genetic bottleneck signature was also tested in population of 2017 using the genetic software Bottelneck 1.2.02^85^. The software measures the temporary excess of heterozygosity that results from a decrease of the effective population size and proposes tests to detect this anomaly^86,87^. Deviations from expected heterozygosity were computed through 1000 permutations, using both the stepwise mutation model (SMM) and the two-phased model of mutation (TPM). One-tailed Wilcoxon sign-rank tests were used to determine whether a population exhibits significant heterozygosity deficit or excess.

## Supplementary information

Supp data Fig 1, 2, Table 1-3

## References

[1] Oerke E-C, Dehne H-W (2004). Safeguarding production—losses in major crops and the role of crop protection. Crop Protec..

[2] Jarvis A, Ramirez-Villegas J, Campo BVH, Navarro-Racines C (2012). Is cassava the answer to African climate change adaptation?. Trop. Plant Biol..

[3] Howeler, R., Lutaladio, N. & Thomas, G. *Save and grow: cassava. A guide to sustainable production intensification*. (FAO, 2013).

[4] Manyong, V. *Impact: The contribution of IITA-improved cassava to food security in Sub-Saharan Africa*. (IITA, 2000).

[5] FAO. FAOstat. *Retrieved Feb* **2014** (2014).

[6] Legg J, French R, Rogan D, Okao‐Okuja G, Brown J (2002). A distinct *Bemisia tabaci* (Gennadius)(Hemiptera: Sternorrhyncha: Aleyrodidae) genotype cluster is associated with the epidemic of severe cassava mosaic virus disease in Uganda. Mol. Ecol..

[7] Jacobson AL, Duffy S, Sseruwagi P (2018). Whitefly-transmitted viruses threatening cassava production in Africa. Current opinion in virology.

[8] Hong Y, Robinson D, Harrison B (1993). Nucleotide sequence evidence for the occurrence of three distinct whitefly-transmitted geminiviruses in cassava. J. Gen. Virol..

[9] Patil B, Legg J, Kanju E, Fauquet C (2015). Cassava brown streak disease: a threat to food security in Africa. J. Gen. Virol..

[10] Monger W, Seal S, Isaac A, Foster G (2001). Molecular characterization of the cassava brown streak virus coat protein. Plant Pathol..

[11] Hillocks R, Raya M, Thresh J (1996). The association between root necrosis and above‐ground symptoms of brown streak virus infection of cassava in southern Tanzania. Int. J. Pest Manag..

[12] Lister R (1959). Mechanical transmission of cassava brown streak virus. Nature.

[13] Legg J, Owor B, Sseruwagi P, Ndunguru J (2006). Cassava mosaic virus disease in East and Central Africa: epidemiology and management of a regional pandemic. Adv. Virus Res..

[14] Hillocks R, Raya M, Mtunda K, Kiozia H (2008). Effects of brown streak virus disease on yield and quality of cassava in Tanzania. J. Phytopathol..

[15] De Barro PJ, Liu S-S, Boykin LM, Dinsdale AB (2011). *Bemisia tabaci:* a statement of species status. Ann. Rev. Entomol..

[16] Boykin Laura M., Shatters Jr. Robert G., Rosell Rosemarie C., McKenzie Cindy L., Bagnall Ruth Ann, De Barro Paul, Frohlich Donald R. (2007). Global relationships of Bemisia tabaci (Hemiptera: Aleyrodidae) revealed using Bayesian analysis of mitochondrial COI DNA sequences. Molecular Phylogenetics and Evolution.

[17] Dinsdale A, Cook L, Riginos C, Buckley Y, De Barro P (2010). Refined global analysis of *Bemisia tabaci* (Hemiptera: Sternorrhyncha: Aleyrodoidea: Aleyrodidae) mitochondrial cytochrome oxidase 1 to identify species level genetic boundaries. Annu. Rev. Entomol..

[18] Legg JP, Fauquet CM (2004). Cassava mosaic geminiviruses in Africa. Plant Mol. Biol..

[19] Davidson EW, Segura BJ, Steele T, Hendrix DL (1994). Microorganisms influence the composition of honeydew produced by the silverleaf whitefly, *Bemisia argentifolii*. J. Insect Physiol..

[20] Maruthi M (2005). Transmission of cassava brown streak virus by *Bemisia tabaci* (Gennadius). J. Phytopathol..

[21] Colvin J, Omongo C, Maruthi M, Otim‐Nape G, Thresh J (2004). Dual begomovirus infections and high *Bemisia tabaci* populations: two factors driving the spread of a cassava mosaic disease pandemic. Plant Pathol..

[22] Polston JE, De Barro P, Boykin LM (2014). Transmission specificities of plant viruses with the newly identified species of the *Bemisia tabaci* species complex. Pest Manag. Sci..

[23] Legg J, Ogwal S (1998). Changes in the incidence of African cassava mosaic virus disease and the abundance of its whitefly vector along south–north transects in Uganda. J. Appl. Entomol..

[24] Holt, J. & Colvin, J. In *Biotic Interactions in Plant-Pathogen Associations* (eds Jeger, M. J. & Spence N. J.) 331–343 (the British Society for Plant Pathology, CABI publishing, 2001).

[25] Legg JP (2014). Spatio-temporal patterns of genetic change amongst populations of cassava *Bemisia tabaci* whiteflies driving virus pandemics in East and Central Africa. Virus Res..

[26] Legg JP (2014). Biology and management of Bemisia whitefly vectors of cassava virus pandemics in Africa. Pest Manag. Sci..

[27] Berry SD (2004). Molecular evidence for five distinct *Bemisia tabaci* (Homoptera: Aleyrodidae) geographic haplotypes associated with cassava plants in sub-Saharan Africa. Ann. Entomol. Soc. Am..

[28] Sseruwagi P (2006). Colonization of non‐cassava plant species by cassava whiteflies (*Bemisia tabaci*) in Uganda. Entomologia experimentalis et applicata.

[29] De la Rúa P, Simon B, Cifuentes D, Martinez‐Mora C, Cenis J (2006). New insights into the mitochondrial phylogeny of the whitefly *Bemisia tabaci* (Hemiptera: Aleyrodidae) in the Mediterranean basin. JZS.

[30] Mugerwa H (2012). Genetic diversity and geographic distribution of *Bemisia tabaci* (Gennadius)(Hemiptera: Aleyrodidae) genotypes associated with cassava in East Africa. Ecol. Evol..

[31] Wosula EN, Chen W, Fei Z, Legg JP (2017). Unravelling the genetic diversity among cassava *Bemisia tabaci* whiteflies using NextRAD sequencing. Genome biology and evolution.

[32] Mbanzibwa D (2011). Simultaneous virus-specific detection of the two cassava brown streak-associated viruses by RT-PCR reveals wide distribution in East Africa, mixed infections, and infections in *Manihot glaziovii*. J. Virol. Methods.

[33] Mugerwa H (2012). Genetic diversity and geographic distribution of *Bemisia tabaci* (Gennadius) (Hemiptera: Aleyrodidae) genotypes associated with cassava in East Africa. Ecol. Evol..

[34] Mouton L (2015). Detection of genetically isolated entities within the Mediterranean species of *Bemisia tabaci*: new insights into the systematics of this worldwide pest. Pest Manag. Sci..

[35] Saleh D, Laarif A, Clouet C, Gauthier N (2012). Spatial and host‐plant partitioning between coexisting Bemisia tabaci cryptic species in Tunisia. Pop. Ecol..

[36] Dalmon A, Halkett F, Granier M, Delatte H, Peterschmitt M (2008). Genetic structure of the invasive pest *Bemisia tabaci*: evidence of limited but persistent genetic differentiation in glasshouse populations. Heredity.

[37] Thierry M (2015). Mitochondrial, nuclear and endosymbiotic diversity of two recently introduced populations of the invasive *Bemisia tabaci* MED species in la Réunion. Insect Cons. Div..

[38] Gauthier N (2014). Genetic structure of Bemisia tabaci Med populations from home‐range countries, inferred by nuclear and cytoplasmic markers: impact on the distribution of the insecticide resistance genes. Pest management science.

[39] Hadjistylli M, Roderick GK, Brown JK (2016). Global population structure of a worldwide pest and virus vector: genetic diversity and population history of the *Bemisia tabaci* sibling species group. PloS one.

[40] Maruthi M (2004). Reproductive incompatibility and cytochrome oxidase I gene sequence variability amongst host‐adapted and geographically separate *Bemisia tabaci* populations (Hemiptera: Aleyrodidae). Syst. Entomol..

[41] Mugerwa H (2018). African ancestry of New World, *Bemisia tabaci*-whitefly species. Scient. Reports.

[42] Van Oosterhout C, Hutchinson WF, Wills DP, Shipley P (2004). MICRO‐CHECKER: software for identifying and correcting genotyping errors in microsatellite data. Mol. Ecol. Notes.

[43] Evanno G, Regnaut S, Goudet J (2005). Detecting the number of clusters of individuals using the software srtucture: a simulation study. Mol. Ecol..

[44] Legg JP (2011). Comparing the regional epidemiology of the cassava mosaic and cassava brown streak virus pandemics in Africa. Virus Res..

[45] Kalyebi A (2018). African cassava whitefly, *Bemisia tabaci*, cassava colonization preferences and control implications. PloS one.

[46] Legg J (1999). Emergence, spread and strategies for controlling the pandemic of cassava mosaic virus disease in east and central Africa. Crop Prot..

[47] Ntawuruhunga, P. & Legg, J. New spread of cassava brown streak virus disease and its implications for the movement of cassava germplasm in the East and Central African region. *USAID, Crop Crisis Control Project C3P* (2007).

[48] Otim-Nape, G., Thresh, J. & Fargette, D. In *Bemisia: 1995, Taxonomy, Biology, Damage, Control and Management* (eds Gerling, D. & Mayer, A. M.) (Elsevier, Intercept, 1996).

[49] Ghosh S, Bouvaine S, Richardson SC, Ghanim M, Maruthi M (2018). Fitness costs associated with infections of secondary endosymbionts in the cassava whitefly species *Bemisia tabaci*. Journal of Pest Science.

[50] Sseruwagi P (2005). Genetic diversity of *Bemisia tabaci* (Gennadius) (*Hemiptera: Aleyrodidae*) populations and presence of the B biotype and a non-B biotype that can induce silverleaf symptoms in squash, in Uganda. Ann. App. Biol..

[51] Ghosh, S., Bouvaine, S. & Maruthi, M. N. Prevalence and genetic diversity of endosymbiotic bacteria infecting cassava whiteflies in Africa. *BMC Microbiol* **15**, 10.1186/s12866-015-0425-5 (2015). 10.1186/s12866-015-0425-5 PMC4434523 25933928

[52] Liu SS (2007). Asymmetric mating interactions drive widespread invasion and displacement in a whitefly. Science.

[53] Delatte H (2006). Microsatellites reveal the coexistence and genetic relationships between invasive and indigenous whitefly biotypes in an insular environment. Genet. Res..

[54] O’Neill S, Giordano R, Colbert A, Karr T, Robertson H (1992). 16S rRNA phylogenetic analysis of the bacterial endosymbionts associated with cytoplasmic incompatibility in insects. PNAS.

[55] Kikuchi Y (2009). Endosymbiotic bacteria in insects: their diversity and culturability. Microbes and Environments.

[56] Thierry M (2011). Symbiont diversity and non‐random hybridization among indigenous (Ms) and invasive (B) biotypes of *Bemisia tabaci*. Mol. Ecol..

[57] Tajebe L (2015). Diversity of symbiotic bacteria associated with *Bemisia tabaci* (Homoptera: Aleyrodidae) in cassava mosaic disease pandemic areas of Tanzania. Ann. App. Biol..

[58] Alicai T (2007). Cassava brown streak disease re-emerges in Uganda. Plant Dis..

[59] Omongo CA (2012). African cassava whitefly, *Bemisia tabaci*, resistance in African and South American cassava genotypes. J. Int. Agricult..

[60] Sseruwagi P, Sserubombwe W, Legg J, Ndunguru J, Thresh J (2004). Methods of surveying the incidence and severity of cassava mosaic disease and whitefly vector populations on cassava in Africa: a review. Virus Res..

[61] Delatte H (2011). Genetic diversity, geographical range and origin of *Bemisia tabaci* (Hemiptera: Aleyrodidae) Indian Ocean Ms. Bull. Entomol. Res.

[62] Kearse M (2012). Geneious Basic: an integrated and extendable desktop software platform for the organization and analysis of sequence data. Bioinformatics.

[63] Rozas J, Sánchez-DelBarrio JC, Messeguer X, Rozas R (2003). DnaSP, DNA polymorphism analyses by the coalescent and other methods. Bioinformatics.

[64] Thompson JD, Higgins DG, Gibson TJ (1994). CLUSTAL W: improving the sensitivity of progressive multiple sequence alignment through sequence weighting, position-specific gap penalties and weight matrix choice. Nucl. Ac. Res..

[65] Posada D (2008). jModelTest: phylogenetic model averaging. Molecular Biology and Evolution.

[66] Ronquist F, Huelsenbeck JP (2003). MRBAYES 3: Bayesian phylogenetic inference under mixed models. Bioinformatics.

[67] Malausa T (2011). High‐throughput microsatellite isolation through 454 GS‐FLX titanium pyrosequencing of enriched DNA libraries. Mol. Ecol. Res..

[68] Atiama M, Delatte H, Deguine J-P (2016). Isolation and characterization of 11 polymorphic microsatellite markers developed for *Orthops palus* (heteroptera: miridae). J. Insect Sci..

[69] Meglécz E (2009). QDD: a user-friendly program to select microsatellite markers and design primers from large sequencing projects. Bioinformatics.

[70] Pritchard JK, Stephens M, Donnelly P (2000). Inference of population structure using multilocus genotype data. Genetics.

[71] Earl DA (2012). Structure Harvester: a website and program for visualizing Structure output and implementing the Evanno method. Cons. Genet Res..

[72] Kopelman NM, Mayzel J, Jakobsson M, Rosenberg NA, Mayrose I (2015). Clumpak: a program for identifying clustering modes and packaging population structure inferences across K. Mol. Ecol. Res..

[73] Jakobsson M, Rosenberg NA (2007). CLUMPP: a cluster matching and permutation program for dealing with label switching and multimodality in analysis of population structure. Bioinformatics.

[74] Rosenberg NA (2004). Distruct: a program for the graphical display of population structure. Mol. Ecol. Res..

[75] Falush D, Stephens M, Pritchard JK (2007). Inference of population structure using multilocus genotype data: dominant markers and null alleles. Mol. Ecol. Res..

[76] Nei M (1978). Estimation of average heterozygosity and genetic distance from a small number of individuals. Genetics.

[77] Weir BS, Cockerham CC (1984). Estimating F‐statistics for the analysis of population structure. Evolution.

[78] Excoffier, L., Laval, G. & Schneider, S. Arlequin (version 3.0): an integrated software package for population genetics data analysis. *E**vol. Bioinformatics* **1** (2005). PMC2658868 19325852

[79] Guo, S. W. & Thompson, E. A. Performing the exact test of Hardy-Weinberg proportion for multiple alleles. *Biometrics*, 361–372 (1992). 1637966

[80] Rousset F (2008). genepop’007: a complete re‐implementation of the genepop software for Windows and Linux. Mol. Ecol. Res..

[81] Fisher RA (1935). The logic of inductive inference. J R Stat Soc.

[82] Goudet, J. Fstat, a Program to Estimate and Test Gene Diversities and Fixation Indices Version 2.9.3. Available from, http://www.unil.ch/izea/softwares/fstat.html [updatedfrom Goudet (1995)] (2002).

[83] R Development Core Team R: A language and environment for statistical computing. R Foundation for Statistical Computing, V., Austria. ISBN 3-900051-07-0, URL, http://www.R-project.org (2008).

[84] Jombart T (2008). adegenet: a R package for the multivariate analysis of genetic markers. Bioinformatics.

[85] Piry S, Luikart G, Cornuet J (1999). BOTTLENECK: a computer program for detecting recent reductions in the effective population size using allele frequency data. J. Hered..

[86] Cornuet JM, Luikart G (1996). Description and power analysis of two tests for detecting recent population bottlenecks from allele frequency data. Genetics.

[87] Luikart G, Cornuet JM (1998). Empirical evaluation of a test for identifying recently bottlenecked populations from allele frequency data. Conserv. Biol..

