# Supplementary material for: What has changed in the outbreaking populations of the severe crop pest whitefly species in cassava in two decades?
Source: Sci Rep. 2019 Oct 15;9:14796. doi: 10.1038/s41598-019-50259-0 (PMC6794263; doi:10.1038/s41598-019-50259-0)
Supplement: Supplementary file 1 — Supp data Fig 1, 2, Table 1-3 [file 41598_2019_50259_MOESM1_ESM.pdf]

Supplementary data

What has changed in the outbreaking populations of the severe crop pest whitefly species in cassava in two decades?

Hadija M. Ally[1,2,3], Hajar El Hamss[4], Christophe Simiand[2], M. N. Maruthi[4], John Colvin[4], Christopher A. Omongo[5], Helene Delatte[2*]

[1]Université de La Réunion Site du CS 92003 97744 Cedex9, 97715, 15 Avenue René Cassin, Sainte-Clotilde, Réunion.

[2]CIRAD, UMR PVBMT, 7 Chemin de l'Irat, Ligne Paradis, 97410, Saint Pierre, La Réunion, France.

[3]Lake Zone Agriculture Research and Development Institute, P.O. Box, 1433, Mwanza, Tanzania.

[4]Natural Resources Institute (NRI), University of Greenwich, Central Avenue, Chatham Maritime, Kent, ME4 4TB, UK.

[5]Root Crops Programme, National Crops Resource Research Institute (RCP-NaCRRI), P.O. Box, 7084, Kampala, Uganda.

*** Corresponding author: Hélène Delatte.** Email: helene.delatte@cirad.fr,

Tel: + 262 262 49 27 35

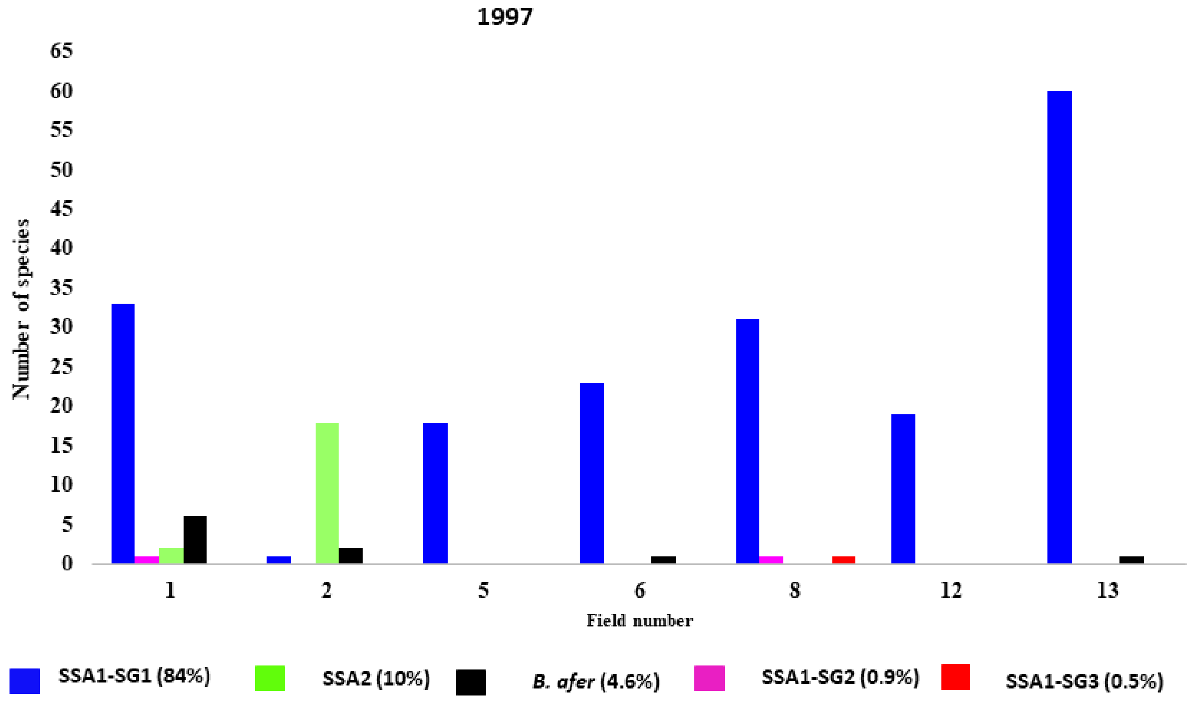

Supp Figure 1: Species distribution per field for samples collected in Uganda 1997

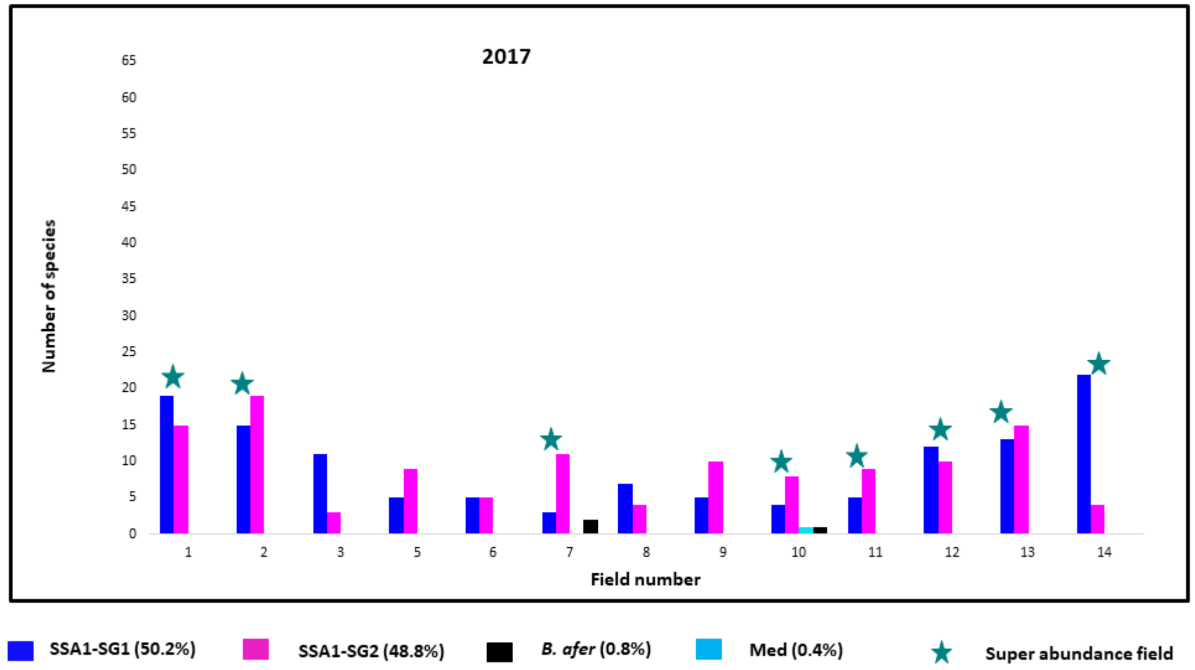

Supp Figure 2: Species distribution per field for samples collected in Uganda 2017

Supp Table 1: Population genetic diversity indices. Population and field number where individuals collected (population/Fn), number of individual sampled (n), mean number of allele per population (NA), (Ar) allele richness, non-biased expected heterozygosity (Hnb), observed heterozygosity (Ho), and fixation indices (Fis) presented together with p value from Hardy-Weinberg equilibrium test, none of the Fis were found significant at $p < 0.05$.

Population/Fno	n	NA	Ar	Ho	Hnb	Fis
SSA1SG1/G2						
F1 - 1997	31	9	6.26	0.35 (0.22)	0.65 (0.29)	0.47
F2 - 1997	2	2	-	0.29 (0.4)	0.51 (0.33)	0.53
F5 - 1997	18	7.83	6.55	0.34 (0.3)	0.66 (0.28)	0.49
F6 - 1997	24	8	6.49	0.37 (0.23)	0.67 (0.26)	0.45
F8 - 1997	32	8.75	6.44	0.36 (0.24)	0.67 (0.24)	0.47
F12 - 1997	19	7.25	6.2	0.32 (0.23)	0.64 (0.29)	0.51
F13 - 1997	57	12.17	6.66	0.4 (0.25)	0.66 (0.28)	0.4
F1 - 2017	15	6	5.29	0.43 (0.24)	0.51 (0.32)	0.17
F2 - 2017	14	4.5	4.22	0.37 (0.26)	0.46 (0.3)	0.21
F3 - 2017	35	7.83	4.94	0.38 (0.27)	0.48 (0.3)	0.22
F5 - 2017	35	8.17	5.36	0.42 (0.28)	0.53 (0.33)	0.21
F6 - 2017	29	7.67	5.44	0.35 (0.2)	0.57 (0.28)	0.38
F7 - 2017	34	8.58	5.86	0.42 (0.23)	0.59 (0.27)	0.3
F8 - 2017	31	8.42	5.4	0.39 (0.24)	0.54 (0.27)	0.27
F9 - 2017	31	8.33	5.41	0.38 (0.25)	0.56 (0.24)	0.32
F10 - 2017	33	8.42	5.74	0.38 (0.22)	0.56 (0.29)	0.32
F11 - 2017	34	8.75	5.91	0.38 (0.24)	0.59 (0.28)	0.35
F12 - 2017	35	8.67	5.46	0.39 (0.2)	0.55 (0.27)	0.3
F13 - 2017	35	8	5.13	0.37 (0.24)	0.51 (0.32)	0.27
F14 - 2017	35	7.58	5.09	0.38 (0.25)	0.51 (0.3)	0.26
SSA2						
F1-1997	3	2.5	2.5	0.22 (0.26)	0.52 (0.27)	0.62
F2-1997	12	4.92	3.08	0.30 (0.28)	0.63 (0.25)	0.54

Supp Table 2. Fst matrix of SSA1 Ugandan populations from 1997 and 2017. All bolded numbers are significant.

		1	2	3	4	5	6	7	8	9	10	11	12	13	14	15	16	17	18	19	20	21	22	23	24	25	26	27	28	29	30	31
F1-SG1-1997	1	0																														
F5-SG1-1997	2	**0.054**	0																													
F6-SG1-1997	3	**0.052**	**0.015**	0																												
F8-SG1-1997	4	**0.032**	**0.02**	**0.023**	0																											
F12-SG1-1997	5	**0.044**	**0.008**	**0.034**	**0.032**	0																										
F13-SG1-1997	6	**0.026**	0.029	**0.016**	0.012	**0.024**	0																									
F1-SG1-2017	7	**0.104**	0.104	**0.125**	**0.149**	**0.116**	**0.074**	0																								
F1-SG2-2017	8	**0.08**	0.06	**0.099**	**0.096**	**0.073**	**0.058**	0.014	0																							
F2-SG1-2017	9	**0.086**	0.096	**0.135**	**0.123**	**0.103**	**0.07**	0.056	-0.001	0																						
F2-SG2-2017	10	**0.098**	0.118	**0.145**	**0.152**	**0.12**	**0.069**	0.039	-0.006	0.011	0																					
F3-SG1-2017	11	**0.102**	0.09	**0.127**	**0.117**	**0.097**	**0.077**	**0.05**	0.012	0.001	0.011	0																				
F5-SG1-2017	12	**0.082**	0.091	**0.113**	**0.112**	**0.083**	**0.068**	**0.081**	0.015	0.003	**0.037**	**0.035**	0																			
F5-SG2-2017	13	**0.078**	0.072	**0.106**	**0.108**	**0.078**	**0.055**	0.027	-0.01	0	-0.011	0.004	0.013	0																		
F6-SG1-2017	14	**0.053**	0.043	**0.084**	**0.068**	**0.061**	**0.041**	0.042	0	0.009	0.032	**0.022**	0.016	0.013	0																	
F6-SG2-2017	15	**0.05**	0.065	**0.095**	**0.065**	**0.068**	**0.038**	**0.058**	0.023	0.013	0.04	**0.033**	**0.024**	0.022	0.013	0																
F7-SG1-2017	16	**0.049**	**0.042**	**0.07**	**0.056**	0.046	0.026	**0.081**	0.01	0.002	0.049	0.026	0.001	0.014	0.003	-0.006	0															
F7-SG2-2017	17	**0.057**	0.065	**0.1**	**0.079**	**0.069**	**0.039**	**0.051**	0.024	0.024	**0.034**	**0.038**	**0.02**	**0.02**	0.017	0.004	0	0														
F8-SG1-2017	18	**0.064**	0.068	**0.085**	**0.076**	**0.057**	**0.038**	0.047	0.016	0.005	0.012	**0.025**	0.023	0.013	0.018	0.014	0.006	0.006	0													
F8-SG2-2017	19	**0.06**	0.082	**0.109**	**0.093**	**0.07**	**0.054**	**0.073**	0.018	-0.003	0.03	**0.019**	0.009	0.014	0.01	-0.001	0	0.01	0.008	0												
F9-SG1-2017	20	**0.042**	0.077	**0.095**	**0.067**	**0.063**	**0.048**	**0.098**	0.033	0.007	**0.052**	**0.048**	0.023	**0.036**	0.032	0.012	-0.001	**0.024**	0.008	-0.003	0											
F9-SG2-2017	21	**0.052**	0.088	**0.104**	**0.091**	**0.074**	**0.045**	**0.079**	**0.043**	0.031	**0.043**	**0.05**	**0.024**	**0.032**	**0.043**	0.024	0.022	**0.022**	-0.003	0.005	0.002	0										
F10-SG1-2017	22	**0.078**	0.082	**0.095**	**0.094**	**0.074**	**0.053**	**0.084**	**0.043**	0.045	**0.07**	**0.039**	0.015	**0.034**	0.031	0.012	0.008	0.012	0.022	0.009	0.028	0.02	0									
F10-SG2-2017	23	**0.062**	0.063	**0.076**	**0.072**	**0.061**	**0.044**	**0.076**	**0.035**	0.027	**0.044**	**0.034**	0.021	**0.026**	0.023	0.007	0.005	0.013	0.009	0.007	0.017	0.013	0.007	0								
F11-SG1-2017	24	**0.059**	0.062	**0.089**	**0.085**	**0.063**	**0.042**	**0.058**	0.014	-0.008	0.025	0.016	0.009	0.01	0.011	0.002	0.003	0.007	-0.004	-0.008	0.008	0.01	0.005	-0.002	0							
F11-SG2-2017	25	**0.045**	0.043	**0.081**	**0.053**	**0.045**	**0.024**	**0.068**	0.03	0.012	**0.043**	**0.035**	**0.025**	**0.026**	0.024	-0.002	0.001	0.005	0.007	0.005	0.015	0.021	0.019	0.012	-0.002	0						
F12-SG1-2017	26	**0.062**	0.078	**0.098**	**0.082**	**0.072**	**0.044**	**0.085**	**0.035**	0.021	0.027	**0.038**	0.011	**0.025**	0.027	0.007	0.005	0.009	-0.001	0.002	0.008	0.004	0.012	-0.002	0	0.011	0					
F12-SG2-2017	27	**0.054**	0.075	**0.11**	**0.092**	**0.086**	**0.053**	**0.058**	0.022	-0.007	0.015	**0.021**	0.017	0.018	0.021	0.012	0.004	0.007	0	0.008	0.01	0.013	0.028	0.016	0.001	0.006	0.005	0				
F13-SG1-2017	28	**0.075**	0.071	**0.107**	**0.1**	**0.075**	**0.04**	**0.059**	0.022	0.023	0.033	**0.031**	0.004	0.02	0.026	0.009	0.009	0.013	-0.001	0.002	0.024	0.01	-0.005	0.013	0.005	0.015	0.002	0.017	0			
F13-SG2-2017	29	**0.082**	0.106	**0.13**	**0.124**	**0.105**	**0.058**	**0.096**	0.029	0.032	**0.036**	**0.03**	0.01	0.022	0.02	0.014	0.014	**0.018**	0.01	0.008	0.025	0.021	0.013	0.016	0.018	**0.027**	-0.002	0.012	0	0		
F14-SG1-2017	30	**0.084**	0.084	**0.122**	**0.11**	**0.095**	**0.065**	**0.068**	0.01	0.001	0.022	**0.02**	0.009	0.014	**0.036**	0.013	0.009	**0.022**	0.007	0.002	0.018	**0.021**	**0.029**	0.012	-0.001	**0.018**	0.006	0.002	**0.021**	0.016	0	
F14-SG2-2017	31	0.05	0.07	**0.106**	**0.1**	**0.071**	0.031	**0.078**	0.014	0.024	0.011	0.024	-0.006	0	0.018	0.017	0.005	0.003	-0.016	-0.003	0.005	-0.009	-0.006	0.002	-0.006	0.006	-0.009	-0.001	-0.013	-0.013	0.018	0

Supp Table 3: Probability values of one-tailed Wilcoxon sign-rank tests to verify significant heterozygosity (H) deficit or excess as expected after recent expansion or reduction of population sizes performed on 2017 samples. Analyses were performed under the single step mutation model (SMM) and the two-phased model of mutation (TPM). Significant values (α=0.05) are in bold.

	**H excess**		**H deficit**	
	**SMM**	**TPM**	**SMM**	**TPM**
**F1**	0.997	0.966	**0.009**	0.083
**F2**	0.998	0.973	**0.007**	0.067
**F3**	1	0.999	**0.001**	**0.002**
**F5**	0.999	0.992	**0.003**	**0.021**
**F6**	0.999	0.998	**0.002**	**0.005**
**F7**	1	0.998	**0.001**	**0.006**
**F8**	1	0.999	**0.001**	**0.002**
**F9**	1	0.998	**0.001**	**0.005**
**F10**	1	0.999	**0**	**0.002**
**F11**	1	0.998	**0.001**	**0.005**
**F12**	1	0.999	**0**	**0.003**
**F13**	1	1	**0**	**0.001**
**F14**	1	0.999	**0.001**	**0.003**
